# Cholesterol in Mitochondrial Diseases—Friend or Foe?

**DOI:** 10.3390/ijms27104353

**Published:** 2026-05-13

**Authors:** Mila Taylor, Michal Halicki, Paul Chazot

**Affiliations:** 1 Department of Biosciences, Durham University, Durham DH1 3LE, UK; mila.taylor@durham.ac.uk (M.T.); michal.halicki.24@ucl.ac.uk (M.H.); 2 Medical Research Council Prion Unit at UCL, Institute of Prion Diseases, University College London, London W1W 7FF, UK

**Keywords:** mitochondria, cholesterol, statin, lipid, dyshomeostasis

## Abstract

Serving as central signalling organelles and hubs of metabolism, mitochondria are essential for cellular homeostasis. Mitochondrial disease can arise from mutations to nuclear or mitochondrial DNA, which result in disruptions to normal mitochondrial function. This generates a suite of rare disorders which are multi-system and often fatal. Variable tissue distribution of mitochondria, alongside a high degree of heterogeneity in associated phenotype, has resulted in an inadequate understanding and characterisation of mitochondrial disease. Addressing this issue is therefore crucial for better clinical management and patient outcomes. Cholesterol dyshomeostasis is a potential pathological hallmark of numerous mitochondrial diseases. Cholesterol is an essential lipid and bioactive compound involved in numerous mitochondrial and cellular processes. A growing number of studies have reported perturbations to cholesterol biosynthesis, cholesterol import, and cholesterol ratios in cell and animal models and individuals with mitochondrial disease, suggesting it could be a unifying feature of these disparate and variable disorders. This review summarises the current experimental evidence for the role of cholesterol dyshomeostasis in mitochondrial disease. It will further discuss reports of statin intolerance, generally attributed to off-target action on mitochondrial structures, in the context of this evidence. Ultimately, the necessity of further integrative clinical and experimental studies exploring the potential of cholesterol dyshomeostasis as a pathological hallmark of mitochondrial disease will be highlighted.

## 1. Introduction

Mitochondrial diseases comprise a heterogeneous group of rare genetic disorders characterised by impaired mitochondrial function. They affect an estimated 1 in 5000 adults [1] and 5 to 15 in 100,000 in the United Kingdom [2]. They arise from pathogenic mutations in either mitochondrial (mtDNA) or nuclear (nDNA) DNA encoding proteins essential for mitochondrial integrity and activity. Such proteins play a role in the maintenance of mitochondrial architecture and membrane potential, ATP generation through oxidative phosphorylation (OXPHOS), mtDNA replication or organisation, transcription, translation, and the import of molecules essential for mitochondrial survival [2,3].

Given the extensive presence of mitochondria in human tissues, mitochondrial diseases are clinically heterogeneous and frequently affect multiple body systems. Symptoms vary largely between individuals and are influenced by many factors, such as the tissue-specific distribution of dysfunctional mitochondria and levels of heteroplasmy. Clinical features can be neurological or non-neurological, encompassing epilepsy, ataxia, dementia, diabetes mellitus, myopathy, and multiple organ failure [2,3]. 

Emerging evidence has implicated cholesterol dyshomeostasis as a unifying factor in the pathology of several mitochondrial diseases. Both compensatory elevations in cholesterol levels and broader disruptions to cholesterol homeostasis have been reported in the context of mitochondrial dysfunction [4,5,6,7]. Notably, clinical reports suggest that individuals with mitochondrial conditions appear to have an elevated sensitivity to statin therapy [8,9]. Statins, or HMG-CoA reductase inhibitors, block an early, rate-limiting step of the mevalonate pathway, thereby reducing de novo synthesis of cholesterol [10]. When considered alongside emerging experimental evidence, these clinical observations may support the possibility that cholesterol plays a role in the pathology of these conditions.

Despite these observations, the role of cholesterol homeostasis in mitochondrial disease pathology remains poorly defined. This review synthesises experimental and clinical evidence supporting the presence of cholesterol dyshomeostasis and examines its potential role in the pathology of mitochondrial diseases.

## 2. Molecular Pathology of Mitochondrial Disease

**Figure 1:**
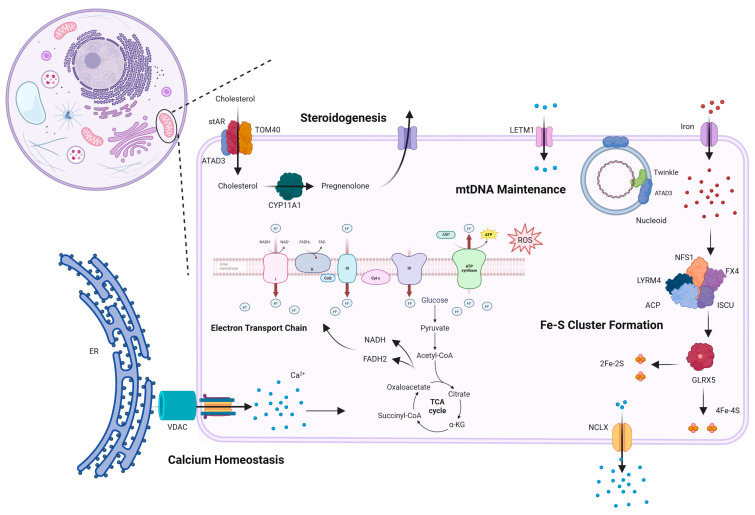
Diagram of mitochondrial function. Highlights several core mitochondrial functions, including calcium homeostasis, oxidative phosphorylation, and steroidogenesis. CYP11A1, cytochrome P450 family 11 subfamily A member; ATAD3, ATPase family ADD domain-containing 3; stAR, steroidogenic acute regulatory; TOM40, translocase of outer mitochondrial membrane 40; VDAC, voltage-dependent anion channel; LETM1, leucine zipper-EF-hand containing transmembrane protein 1. Created in Biorender. Mila Taylor. (2026).

Mitochondria perform numerous essential functions (Figure 1). Their ubiquity means that dysfunction has widespread negative effects. Accordingly, mitochondrial diseases affect a multitude of systems and exhibit substantial clinical variability, ranging from severe and fatal presentations to milder disorders with delayed onset [2,3]. Despite this diversity, certain molecular and phenotypic characteristics remain consistent across mitochondrial diseases.

Dysfunction of oxidative phosphorylation (OXPHOS) is a defining feature of mitochondrial disease and results in impaired ATP production and increased oxidative stress due to elevated mitochondrial reactive oxygen species (mtROS) generation [3,11,12]. These defects are frequently accompanied by loss of mitochondrial membrane potential, impaired protein and metabolite import and export, secondary electron transport chain (ETC) dysfunction, and altered mitochondrial fission and fusion dynamics [3]. Reduced ATP availability may compromise lysosomal function, impairing autophagy-based removal of damaged mitochondria and permitting their accumulation, further exacerbating cellular pathology [13,14]. Collectively, these defects limit the mitochondria’s capacity to meet cellular energy demands and can propagate dysfunction at the tissue and organ levels.

Clinical presentation is highly heterogeneous, with common clinical symptoms including developmental delay, seizures, hypotonia, visual and auditory impairment, and stroke or stroke-like episodes [3]. Mapping between genotype and phenotype remains challenging, as mutations in over 300 genes can produce distinct clinical outcomes, and different clinical symptoms may arise from the same mutation [1,15]. Identifying molecular features that transcend individual genotypes and unify diverse phenotypes is therefore a central goal. Cholesterol homeostasis, recurrently perturbed in several mitochondrial disorders, is crucial for maintaining membrane integrity, facilitating organelle communication, and regulating metabolism, thereby positioning it as a potential contributor to mitochondrial dysfunction. The following section examines the importance of cholesterol for mitochondrial health, reviews evidence linking cholesterol dyshomeostasis to mitochondrial disease, and explores its mechanistic relevance to pathology.

## 3. Evidence for Changes to Cholesterol Homeostasis in Mitochondrial Disease

### 3.1. Role of Cholesterol in Health and Disease

Cholesterol has a critical role in maintaining membrane fluidity, permeability, and curvature, production of steroid hormones, bile acids and lipoproteins, and signal transduction through lipid raft formation [16,17]. Disruption of cholesterol homeostasis has been implicated in multiple conditions, such as cardiovascular diseases, cancers, and neurodegeneration [17,18].

Adequate management of cholesterol is important intracellularly and within individual organelles. Mitochondria require carefully regulated cholesterol to accomplish several key functions. The first and rate-limiting step of steroidogenesis, involving conversion of cholesterol to pregnenolone by CYP11A1, occurs within the mitochondria [19,20]. Twinkle helicase, a major replication protein involved in mtDNA replication, associates with mtDNA-containing nucleoids through interaction with cholesterol-rich replication platforms. High cholesterol content within these platforms allows formation of ER–mitochondrial junctions, providing important membrane architecture [21]. Perturbations to cholesterol homeostasis in either of these scenarios can result in severe pathologies, like neurological syndromes and metabolic disorders [20,21]. Excess cholesterol can also interfere with mitochondrial bioenergetics, elevating ROS production and disrupting biophysical membrane properties, as exemplified in diseases such as Niemann-Pick Type C, Alzheimer’s, and non-alcoholic fatty liver disease [22].

Inducing mitochondrial dysfunction through pharmacological treatment can alter cholesterol homeostasis. Inhibition of ETC complex I in primary fibroblasts decreases cholesterol intermediates, downregulates expression of cholesterol metabolising genes like *SREBF2* and *HMGCR*, and encourages proteolytic processing of existing sterol sensors to prevent their activation. Intracellular cholesterol increases in response, indicating a potential redistribution and highlighting the importance of maintaining sufficient intracellular amounts [23]. Primary fibroblasts from patients with existing mitochondrial conditions also display cholesterol dyshomeostasis, highlighting a potential link between mitochondrial disease and cholesterol homeostasis [4,5,7].

### 3.2. Cholesterol in Mitochondrial Disease

#### 3.2.1. Cholesterol in ATAD3-Related Pathologies

ATPase family ADD domain-containing 3 (ATAD3) plays a central role in mitochondrial structure and function. It is implicated in multiple mitochondrial processes, including mtDNA maintenance through its association with mtDNA-containing nucleoids [24,25,26], cholesterol import for steroidogenesis through association with the steroidogenic acute regulatory protein (stAR) [27,28], cristae organisation [4,29], and mitochondrial fission and fusion through interaction with mitofusin 1 and 2 [25,30] (Figure 1). Consistent with these functions, ATAD3 depletion disrupts nucleoid integrity and mtDNA metabolism [24,30,31], impairs mitochondrial protein synthesis [29], depletes mtDNA [32], and alters cholesterol metabolism [4,29,31,32].

Clinically, pathological variants of ATAD3 give rise to a broad spectrum of phenotypic manifestations. Neurological presentations include cerebellar atrophy, axonal neuropathy, and optic neuropathy, while syndromic manifestations such as cardiomyopathy and seizures have also been reported. The severity of disease manifestation varies depending on the underlying *ATAD3* mutations, which may involve biallelic deletions in the *ATAD3* gene cluster or gene fusions mediated by homologous recombination, such as the *ATAD3A/C* fusion [4,29,31].

Emerging evidence has positioned cholesterol dyshomeostasis as a key feature of ATAD3-related pathologies. Notably, loss of sufficient cellular and mitochondrial cholesterol has been suggested as a unifying pathological feature in these disparate mitochondrial disorders. Fibroblasts derived from individuals harbouring different mutations in the *ATAD3* gene cluster have elevated free cholesterol, alongside aberrant mtDNA organisation (Table 1) [4,29,31,33]. These cellular phenotypes are accompanied by transcriptional changes, including upregulation of genes involved in cholesterol biosynthesis [31,33] and downregulation of genes mediating cholesterol efflux [4], collectively indicating changes to cholesterol homeostasis.

Mechanistic studies support a role for ATAD3 in cholesterol trafficking. Genetic ablation of the *Atad3* gene in mouse skeletal muscle reduced ER synthesis of cholesterol esters (CEs) and decreased the total CE/free cholesterol ratio, indicative of dysfunctional cholesterol trafficking and likely mitochondrial cholesterol deficiency [32]. Pharmacological manipulation of cholesterol levels illustrated the significance of cholesterol for mtDNA maintenance in patient-derived fibroblasts harbouring *ATAD3* mutations. Reduction in intracellular cholesterol through a cholesterol transport inhibitor or pravastatin exacerbated pathological mtDNA aggregation, increasing the disparity between control and ATAD3-deficient fibroblasts [31]. These findings suggest that elevated cholesterol levels may represent a compensatory response.

This hypothesis was reinforced by Munoz-Oreja et al. (2024), who demonstrated that *Drosophila* carrying an orthologous *Atad3^R472C^* mutation exhibit increased dietary dependence on cholesterol [4]. A modified high-sugar diet lacking other components of the standard Drosophila laboratory diet significantly reduced pupal and fly viability. In contrast, supplementation of this diet with cholesterol markedly rescued survival of both pupae and adults. Notably, this improvement occurred despite membrane cholesterol aggregation and increased lysosomal abundance in both patient-derived fibroblasts and *Drosophila* mutants, features that may contribute to deleterious lysosomal insufficiency.

Collectively, these findings support a model in which ATAD3 dysfunction impairs cholesterol trafficking, resulting in insufficient mitochondrial cholesterol availability. Heightened sensitivity to cholesterol perturbation suggests a critical role for cholesterol in ATAD3-related pathogenesis, with elevated cholesterol levels potentially reflecting compensatory metabolic reprogramming that partially mitigates mitochondrial dysfunction [4,31].

#### 3.2.2. Cholesterol in Leigh Syndrome

Emerging evidence suggests that cells harbouring mutations causative of Leight syndrome (LS) exhibit cholesterol dyshomeostasis, which appears to play a role in subsequent pathology. LS is the most common childhood manifestation of a mitochondrial disorder, caused by mutations in more than 75 nuclear and mitochondrial genes. It is characterised by neurological presentation, with neurodevelopmental regression, motor symptoms, epilepsy and encephalopathy [2]. Furthermore, perturbations of cholesterol homeostasis have been associated with multiple mutations causing LS (Table 1). One of the most common mutations associated with LS is a loss-of-function mutation in *SURF1*, a nuclear gene encoding a factor involved in mitochondrial C-IV assembly [40]. Menacho et al. (preprint) showed that neural progenitor cells (NPCs) carrying a homozygous mutation in *SURF1* exhibited lower membrane cholesterol content compared to control NPCs. Treatment of LS NPCs with Sertaconazole or Talarozole significantly elevated membrane cholesterol, and elicited an amelioration of the disease phenotype by improving cellular metabolic profile and enhancing neuromorphogenesis [35]. Pesini et al. (2025) investigated neurons induced from primary fibroblasts from patients with LS carrying pathological mutations in the *PDSS2* gene, which encodes decaprenyl diphosphate synthase involved in (CoQ10) synthesis [34]. They reported a reduction in cholesteryl esters (albeit not significant) and that coenzyme Q10 decreased levels of proteins involved in cholesterol biosynthesis (such as HMGCR) and increased levels of proteins involved in cholesterol efflux (such as ABCA1) [34].

In contrast, genes involved in cholesterol biosynthesis were shown to be upregulated in the cerebellum and hippocampus of mice carrying a knockout of the *Nsduf4* gene, which is associated with C-I deficiency and LS [36]. Intriguingly, research using primary fibroblasts from patients with complex I deficiency suggested a mechanism where increased synthesis of cholesterol consumes elevated NADPH associated with cellular redox imbalance. Promoting cholesterol efflux through stimulation of ABCA1 activity increased total cellular cholesterol concentration in vitro, and it also decreased redox stress in primary cells from patients carrying LS mutations. Further, increasing cholesterol efflux with fenofibrate in *Nsduf4* KO mice extended the mice’s lifespan and improved motor symptoms. These effects appeared to have been associated with elevated cholesterol biosynthesis [7].

Of note, fibrates were previously shown to improve symptoms of mitochondrial disorders in murine models, including mice carrying the *Nsduf4* mutation. However, a high-fat diet alone was not sufficient to induce such effects, which suggests fibrate-specific effects [41]. Indeed, if cholesterol biosynthesis consumes elevated NADPH, which is pathological in LS [7], dietary supplementation of cholesterol would not exert an effect. As such, if modulating cholesterol homeostasis is indeed therapeutic, targeting specific cellular pathways, such as cholesterol efflux or biosynthesis, might be necessary to exert beneficial effects in mitochondrial disease. Alternatively, fibrates might target other processes, for instance, mitochondrial biogenesis, to improve mitochondrial disease [42]. However, evidence exists for the improvement of symptoms in the absence of mitochondrial biogenesis in mouse models of mitochondrial disease [41,43].

Leigh syndrome French Canadian (LSFC) is a variant of LS caused by a founder mutation in the *LRPPRC* gene, which encodes an RNA-binding protein stabilising transcripts of mtDNA. Metabolic profiling from the plasma of patients with LSFC revealed elevated serum total cholesterol/HDL ratio and decreased HDL cholesterol compared to healthy controls [38]. Further studies using lipidomic profiling of plasma from the same cohort of patients revealed significantly elevated 1 cholesteryl ester [37]. These results suggest an altered cholesterol profile in LSFC. In vivo, Cuillerier et al. (2017) showed that mice lacking LRPPRC in hepatocytes exhibited impaired mitochondrial ROS handling and a remodelled lipidomic profile of membranes in mitochondria from hepatocytes [39]. Authors argued that a significant loss of total mitochondrial membrane cholesterol and an increased amount of polyunsaturated fatty acids can affect membrane properties, leading to decreased diffusion of hydrogen peroxide, thereby contributing to pathology [39]. 

Altogether, these results indicate that cholesterol perturbation is associated with LS.

Despite converging clinical manifestations, the specific effects of LS on cholesterol are diverse, even among the same genotypes, potentially reflecting tissue heterogeneity characteristic of mitochondrial disease. However, as illustrated in the above studies, current efforts often have inconsistent or non-uniform measures of cholesterol, confounding the ability to compare or group genotype-specific effects. Identification of certain patterns of cholesterol dyshomeostasis, such as reduced total cholesterol in a specific genotype, could allow for more concrete correlations between genotype and cholesterol outcomes. Table 1 compares different effects of distinct LS mutations on cholesterol homeostasis.

#### 3.2.3. Cholesterol in Other Mitochondrial Diseases

Currently, many studies investigating the role of cholesterol in mitochondrial disease are focused on LS and ATAD3-associated disease. However, evidence for perturbed cholesterol homeostasis has been associated with other mitochondrial disease-causing mutations. One study investigated neurons derived from patient primary fibroblasts. Cells with a mutation in the *APTX* gene, which causes ataxia with oculomotor apraxia type 1, had decreased cholesteryl ester levels but increased expression of proteins involved in cholesterol biosynthesis (HMGCR and SREBP2) and cholesterol efflux (ABCA1). In contrast, cells from patients with a mutation in the *COQ2* gene associated with primary CoQ10 deficiency exhibited decreased HMGCR and ABCA1 expression [34]. Toshima et al. (2024) reported similar downregulation of genes in mice KO of a gene involved in mitochondrial cardiomyopathy [6]. Mice with neuron-specific KO of this *C1qbp* gene also exhibited decreased brain expression of several genes relevant to cholesterol biosynthesis, including SREBP2 and HMGCR [6]. Primary fibroblasts derived from patients with mitochondrial neurogastrointestinal encephalomyopathy (MNGIE), but not with mitochondrial encephalomyopathy, lactic acidosis, and stroke-like episodes (MELAS), also exhibited reduced expression of cholesterol biosynthesis genes and had significantly lowered total cholesterol levels [5].

Metabolic dysfunction-associated steatotic liver disease (MASLD), formerly non-alcoholic fatty liver disease, encompasses a cluster of conditions associated with dyslipidaemia and metabolic and mitochondrial dysfunction. MASLD has become increasingly prevalent since the 1980s, with ~30% of the population affected worldwide. MASLD is generally progressive, with early stages more readily treatable or manageable. Stages include steatosis, metabolic-associated steatohepatitis (MASH), fibrosis, cirrhosis, and hepatocellular carcinomas. Development of MASLD involves the interaction between varied factors, including genetics, environment, and lifestyle [44,45].

The pathogenesis of MASLD involves the accumulation of certain fats, like free fatty acids (FFAs), diacylglycerols, and ceramides, within hepatocytes. This impairs crucial hepatic processes, causes hepatic injury and inflammation, and increases risk for a variety of co-morbid symptoms and syndromes [45,46,47]. Mitochondrial dysfunction can act as a driver or result of hepatic lipid accumulation and promotes ROS production, inflammation, lipid peroxidation, cytokine release, and cellular death. A specific example involves impaired fatty acid oxidation (FAO), which is considered central to the pathogenesis of MASLD and is highly implicated in progression of MASH to more advanced disease states [44,46]. More generally, dyslipidaemia has been shown to increase mitochondrial DNA damage and overarching mitochondrial dysfunction [48,49], illustrating a link between dysregulated lipid homeostasis and mitochondrial function.

Though different than mitochondrial disease-related dysfunction and dyshomeostasis, the direct role of mitochondrial dysfunction in MASLD, a condition of dyslipidaemia, highlights another mechanism through which cholesterol dyshomeostasis and mitochondrial dysfunction may be linked.

Overall, a large body of emerging evidence points to the role of altered cholesterol homeostasis in several mitochondrial diseases (Table 1). Interestingly, the exact perturbation of cholesterol metabolism differs not only between distinct mitochondrial diseases but also between different mutations causing the same disease, for instance, Leigh Syndrome. Further investigations are necessary to improve the understanding of this growing area of interest. 

## 4. Adverse Effects of Statins in Mitochondrial Disease—Potential Role of Cholesterol?

Experimental evidence suggests that perturbed cholesterol homeostasis is present in multiple mitochondrial diseases and might play a role in disease pathology. While clinical research investigating this relationship is limited, several reports have described exacerbation of mitochondrial disease during statin therapy. This evidence could further reinforce the importance of cholesterol homeostasis in mitochondrial disease.

**Figure 2:**
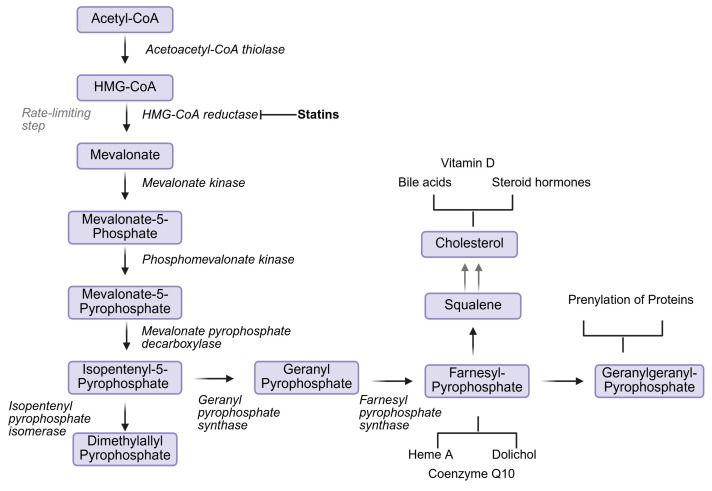
Mevalonate pathway progression, including relevant intermediaries, enzymes, products, and statins (HMG-CoA reductase inhibitors) mode of action. The early inhibition by statins results in a reduction in all downstream products, not only cholesterol. This includes mitochondrially important compounds like Heme A and CoQ10. Created in Biorender. Mila Taylor. (2026).

Statins, or HMG-CoA reductase inhibitors, are among the most prescribed medications [50]. Numerous studies have found statin therapy to be instrumental in reducing cardiovascular events, cardiovascular mortality, and total mortality in patients with elevated blood cholesterol or coronary artery disease [51,52]. They further reduce progression of atherosclerosis, improve endothelial function, reduce vascular inflammation, and reduce platelet adhesion and thrombosis [51,53]. Statins’ cholesterol-lowering effect is attributed to the inhibition of the mevalonate pathway (Figure 2), preventing cholesterol biosynthesis and increasing uptake and degradation of low-density lipoproteins (LDL) in hepatocytes.

However, the widespread action of statins may also facilitate a number of adverse effects (AEs). The mevalonate pathway, which is inhibited by statins, is a crucial metabolic pathway which produces many essential bioactive molecules, both as final products and as intermediaries. These molecules have important roles in protein modification, intracellular signalling, cell growth, gene expression, and cytoskeletal assembly. Further, final pathway products, like cholesterol and Coenzyme Q10 (CoQ10), are indispensable for physiological function. Cholesterol is a precursor to bile acids, lipoproteins, and steroid hormones [17]. CoQ10 is a primary antioxidant, preventing lipid peroxidation, and a key component in the OXPHOS system, acting as a mobile electron carrier [54,55]. Therefore, inhibition of HMG-CoA reductase by statins early in the pathway reduces biologically relevant intermediaries and final products. This reduction, particularly in individuals who may have underlying conditions, can result in statin intolerance or statin AEs [9,17]. 

Though a variety of potential mechanisms may contribute to statin AEs, a mitochondrial basis has been repeatedly implicated in their development [17]. Mitochondrial defects predispose to issues with statins [56], and statins predispose to mitochondrial defects [57,58]. Certain studies have reported reductions in CoQ10 and heme-A, mitochondrially important mevalonate products, as a primary driver. As mentioned, CoQ10 is crucial for OXPHOS function and prevention of lipid peroxidation [54,55]. Commonly, CoQ10 reductions are already present in patients with mitochondrial dysfunction, either due to primary CoQ10 biosynthesis defects or secondary accelerated degradation of CoQ10 following ETC dysfunction and increased ROS [55]. When combined with statins, which inhibit CoQ10 synthesis, patients with existing, disease-driven CoQ10 deficiencies are significantly more likely to develop statin AEs. However, several other AEs have remained unexplained [17,55]. Further, statin intolerance effects, such as myopathy, rhabdomyolysis, neurological symptoms, and myalgia, exhibit variable reversibility and variable presence in individuals with mitochondrial disease [17,57].

Ultimately, statins are advised to be prescribed with caution in mitochondrial disorders. Potential side effects and creatine kinase levels (indicative of muscle damage) should be closely monitored [58,59]. Case reports have associated exacerbation of mitochondrial disease by statin therapy in patients carrying the m.3243A>G mutation in the MT-TL1 gene, causative of MELAS. Chariot et al. (1993) reported that treatment with simvastatin revealed previously unnoticed MELAS syndrome (later confirmed by the presence of the m.3243A>G mutation), with rhabdomyolysis and neurological symptoms, which gradually decreased following simvastatin cessation [60]. Another patient showed deterioration and progression of MELAS symptoms (e.g., focal seizures, lactic acidosis, and other neurological symptoms) coinciding with pravastatin and simvastatin treatment. Treatment with other lipid-lowering drugs, gemfibrozil, fenofibrate, and ezetimibe, following cessation, did not exacerbate the condition but rather improved stroke-like and bowel obstruction episodes [61]. 

Other case reports described more limited symptoms of statin intolerance in patients with mitochondrial disease. In a patient with the MELAS mutation, a short course of treatment with lovastatin and atorvastatin resulted in myalgia, despite not decreasing total cholesterol levels [62]. Similar responses occurred in a patient with MELAS [63] and a patient with a MT-CO1 gene mutation [64]. Several other reports linked musculoskeletal AEs during statin therapy with probable (suggested by family history or muscle biopsy) but genetically unconfirmed mitochondrial disease [65,66,67]. Tsivgoulis et al. (2006) described a case of rhabdomyolysis, with fatigue and muscle weakness persisting even after atorvastatin treatment was discontinued [66].

In all, the rationale for statin prescription with caution is demonstrated. Though the effects of statins on mitochondrial pathways have been proposed to explain a number of these AEs, several cases and symptoms remain unexplained. This is particularly relevant when considering non-statin cholesterol-lowering medication, which function through alternative pathways. Though possessing more favourable safety profiles, which does align with off-target mitochondrial effects of statins, AEs may still arise. Further, many individuals unable to tolerate these alternative methods of cholesterol reduction could also not tolerate statins. This raises the possibility that an alternate underlying factor may be generating some of the AEs in both statin medications. 

Ezetimibe is a generally well tolerated lipid-lowering agent, which selectively impairs intestinal cholesterol absorption through inhibition of sterol transporter Niemann-Pick C1-Like-1 (NPC1L1) at the brush border of the small intestine [68]. This results in increased blood cholesterol clearance, reduced hepatic cholesterol storage, and ultimately facilitates reductions in LDL-C (approximately 13–20%) [68,69]. Widespread clinical trials support a favourable safety profile, particularly for individuals who experience HMG-CoA-intolerances [68,70]. However, isolated AEs have been reported in several publications, through mechanisms which are poorly understood and could intersect with mitochondrial function and physiology. 

Musculoskeletal symptoms, such as myalgia and arthralgia, followed by gastrointestinal symptoms, such as nausea, diarrhoea, and abdominal pain, are the most common AEs reported [68,71]. Several case studies have presented rare and more severe AEs, including reversible rhabdomyolysis [70,72], progressive visual field loss aligned with rod–cone dysfunction [68], and worsening myopathy in a patient with McArdle disease [73]. Notably, a patient with McArdle disease, a metabolic disorder of glycogen storage which primarily impacts skeletal muscle, presented with myopathy, exercise intolerance, and extreme fatigue. This coupled with elevated creatine kinase (CK) indicative of muscle damage [73,74]. Though not a mitochondrial disorder, McArdle disease does impact the musculoskeletal system, similar to mitochondrial diseases. The majority of patients presented with similar biomarkers to HMG-CoA myopathies, including elevated CK and myalgia. Several of the patients had additionally reported earlier statin intolerance [68,70,72,73].

The presence of adverse reactions despite the alternative mechanism of cholesterol reduction may suggest an underlying component contributing to AEs, in the presence or absence of mevalonate pathway inhibition. Currently, this component is unidentified. However, when considered with Section 3.2, which suggests elevated cholesterol as compensatory in certain mitochondrial diseases, there is a tentative case for reductions in total cholesterol to generate AEs, under specific conditions (e.g., mitochondrial disease). Further, the elevated cholesterol present within these individuals may relate to underlying mitochondrial disease. Though current evidence only supports links in vitro and in non-mammalian systems (D. melanogaster), investigations in mammalian systems could determine if this link is widespread. As reductions in total cholesterol in both in vitro and fly systems resulted in less favourable disease outcomes [4,33], a similar phenomenon in humans could explain some of the statin AEs and intolerances. Mitochondrial dysfunction predisposes individuals to statin intolerance [9,56]; reductions in total cholesterol could be implicated in some of this intolerance. The link between non-statin lipid-lowering medication intolerance and mitochondrial disease has also been little investigated. If reductions in cholesterol can indeed be deleterious in certain mitochondrial diseases, the uncertain mechanism behind the intolerance could be partially explained. Further, regardless of links, exploration into mitochondrial disease being a potential contraindication for lipid-lowering medication would allow better patient treatment and outcomes. In all, further research in these areas would greatly improve understanding of cholesterol-lowering medication, mitochondrial disease, and cholesterol itself. 

## 5. Conclusions

Mitochondria, with their own DNA (mtDNA) and RNA (mtRNA), are crucial for key physiological functions such as cellular energy metabolism, apoptosis, and redox balance. Mitochondrial dysfunction can cause a series of hereditary diseases, collectively called mitochondrial disorders. A growing body of evidence points to the role of altered cholesterol homeostasis in several mitochondrial diseases. The precise perturbation of cholesterol metabolism differs not only between distinct mitochondrial diseases but also between different mutations causing the same disease.

## Figures and Tables

**Table 1:** Summary of evidence for cholesterol dyshomeostasis in different mitochondrial diseases.

Mitochondrial Disease	Gene	Model	Effect on Cholesterol	Citation
ATAD3-related pathologies	*ATAD3*	Patient-derived fibroblasts with a heterozygous *ATAD3A* variant and *Drosophila* carrying orthologous *Atad3^R472C^* mutation	↑ free cholesterol ↓ Expression of proteins involved in cholesterol efflux (including *ABCA1* and *ABCG1*) ↑ membrane-bound cholesterol levels ↑ reliance on dietary cholesterol	[4]
		Patient-derived fibroblasts, either with NAHR-mediated ATAD3A/C fusion gene lacking functional residues or biallelic ATAD3 cluster deletions	↑ free cholesterol	[29]
		Skeletal muscle-specific *Atad3* cKO mice	↓ cholesterol esters synthesized within ER ↑ dietary cholesterol esters ↓ cholesterol esters/free cholesterol ratio	[32]
		Patient-derived fibroblasts with biallelic deletions in *ATAD3* gene cluster	↑ free cholesterol ↑ expression of genes involved cholesterol biosynthesis pathway (such as *SREBF2* and *HMGCS1*) and cholesterol efflux (*ABCA1*) • Decreasing cholesterol levels with cholesterol trafficking inhibitor or pravastatin increased mtDNA pathology	[31]
		Patient-derived fibroblasts with biallelic *ATAD3A* variants	↑ free cholesterol ↑ expression of genes involved in cholesterol biosynthesis pathways	[33]
Primary CoQ10 deficiency and Leigh Syndrome	*PDSS2*	Patient-derived fibroblasts	↓ cholesteryl esters (trend) ↓ expression of proteins involved in cholesterol biosynthesis (HMGCR) = Level of SREBP2 ↑ expression of proteins involved in cholesterol efflux (ABCA1)	[34]
Leigh Syndrome	*SURF1*	Neural progenitor cells with homozygous *SURF1* mutation	↓ membrane cholesterol • Increasing membrane cholesterol associated with ameliorated disease phenotype	[35]
	*NSDUF4*	Patient-derived fibroblasts and *Nsduf4* KO mice	• Increasing cholesterol efflux and biosynthesis improved phenotype and increases lifespan of *Nsduf4* KO mice	[7]
		*Nsduf4* KO mice	↑ Upregulated cholesterol biosynthesis pathways in cerebellum and hippocampus	[36]
Leigh Syndrome French Canadian	*LRPPRC*	Plasma from patients	↑ 1 cholesteryl ester	[37]
		Plasma from patients	↑ LDL cholesterol ↓ HDL cholesterol ↑ Total cholesterol/HDL cholesterol ratio	[38]
		Hepatocyte-specific *Lrpprc* cKO mice	↓ mitochondrial membrane cholesterol	[39]
Ataxia with Oculomotor Apraxia Type 1	*APTX*	Neurons derived from patient fibroblasts	↓ cholesteryl esters ↑ expression of proteins involved in cholesterol biosynthesis (HMGCR and SREBP2) ↑ expression of proteins involved in cholesterol biosynthesis cholesterol efflux (ABCA1 and ABCG1)	[34]
Primary CoQ10 deficiency	*COQ2*		↓ expression of proteins involved in cholesterol biosynthesis (HMGCR) = Level of SREBP2 ↓ expression of proteins involved in cholesterol biosynthesis cholesterol efflux (ABCA1 and ABCG1)	
Mitochondrial Cardiomyopathy	*C1QBP*	Neuron-specific *C1qbp* cKO mice	↓ brain expression of genes involved in cholesterol biosynthesis (including *Srebf2*, *Hmgcr*, and *Hmgcs1*)	[6]
MNGIE	*TYMP*	Patient-derived fibroblasts	↓ total cholesterol ↓ expression of protein involved in cholesterol biosynthesis (SREBP1 and SREBP2) and efflux (ABCA1)	[5]
MELAS	*MT-TL1 (m.3243A > G)*		= total cholesterol	

↓ decrease; ↑ increase; = no change; MELAS, mitochondrial encephalomyopathy, lactic acidosis, and stroke-like episodes; MNGIE, mitochondrial neurogastrointestinal encephalomyopathy; *ATAD3*, ATPase family AAA domain-containing protein 3; *SURF1*, SURF1 cytochrome c oxidase assembly factor; *PDSS2*, Prenyl Diphosphate Synthase Subunit 2; NADH dehydrogenase [ubiquinone] iron-sulphur protein 4, mitochondrial; *LRPPRC*, leucine-rich PPR motif-containing protein, mitochondrial; *APTX*, Aprataxin; *COQ2*, para-hydroxybenzoate-polyprenyltransferase; *C1QBP*, complement C1q binding protein; *TYMP*, thymidine phosphorylase; MT-TL1, mitochondrially encoded tRNA-Leu (UUA/G) 1; SREBP2, sterol-responsive element-binding protein 2; HMGCR, 3-hydroxy-3-methylglutaryl-CoA reductase; Hmgcs1, 3-hydroxy-3-methylglutaryl-CoA synthase 1; ABCA1 and ABCG1, ATP-binding cassette transporter A1 and G1.

## Data Availability

No new data were created or analyzed in this study. Data sharing is not applicable to this article.

## Abbreviations

The following abbreviations are used in this manuscript: mtDNA, mitochondrial DNA; (OXPHOS) oxidative phosphorylation; ETC, electron transport chain CYP11A1; ATAD3, ADD domain-containing 3; (CoQ10) coenzyme Q10; NPCs, neural progenitor cells; NADPH, nicotinamide adenine dinucleotide phosphate; ABCA1 and ABCG1, ATP-binding cassette transporter A1 and G1; NPC1L1 transporter, Niemann-Pick C1-Like-1; MELAS, Mitochondrial Encephalomyopathy, Lactic Acidosis, and Stroke-like episodes; MNGIE, Mitochondrial neurogastrointestinal encephalomyopathy; *ATAD3*, ATPase family AAA domain-containing protein 3; *SURF1*, SURF1 cytochrome c oxidase assembly factor; *PDSS2*, Prenyl Diphosphate Synthase Subunit 2; NADH dehydrogenase [ubiquinone] iron-sulfur protein 4, mitochondrial; *LRPPRC*, Leucine-rich PPR motif-containing protein, mitochondrial; *APTX*, Aprataxin; *COQ2*, para-hydroxybenzoate-polyprenyltransferase; *C1QBP*, Complement C1q Binding Protein; *TYMP*, thymidine phosphorylase; MT-TL1, mitochondrially encoded tRNA-Leu (UUA/G) 1; SREBP2, sterol-responsive element-binding protein 2; HMGCR, 3-hydroxy-3-methylglutaryl-CoA reductase; Hmgcs1, 3-hydroxy-3-methylglutaryl-CoA synthase 1; CK, Creatine Kinase.
